# Factors associated with non-adherence to social distancing rules during the COVID-19 pandemic: a logistic regression analysis

**DOI:** 10.1186/s12889-021-10379-7

**Published:** 2021-02-13

**Authors:** Stephen Hills, Yolanda Eraso

**Affiliations:** 1 Guildhall School of Business and Law, London Metropolitan University, 166-220 Holloway Rd, London, N7 8DB England; 2 School of Social Professions, London Metropolitan University, 166-220 Holloway Rd, London, N7 8DB England

**Keywords:** COVID-19, London, Pandemic, Social distancing, Behaviours, Non-adherence

## Abstract

**Background:**

On March 23, 2020, the government of the United Kingdom told the British people to stay home, an unprecedented request designed to limit the spread of the COVID-19 virus and stop the National Health Service from being overwhelmed.

**Methods:**

This study undertook a cross-sectional design to survey a convenience sample of 681 residents of North London on their social distancing (SD) behaviours, demographics, housing situation, politics, psychology and social support using an online questionnaire. Logistic regression was used to measure the associations between these explanatory factors and non-adherence to all SD rules and intentional non-adherence to SD rules.

**Results:**

The vast majority (92.8%) of participants did not adhere to all SD rules and nearly half (48.6%) engaged in intentional non-adherence of rules. The odds of not adhering to all SD rules increased if a participant was not identified as highly vulnerable to COVID-19 [OR = 4.5], had lower control over others’ distancing [OR = .724], had lower control over responsibilities for which coming into contact with others was unavoidable [OR = .642], and if SD behaviours were reported after lockdown was first relaxed [OR = .261]. The odds of intentionally not adhering to SD rules increased if a participant had a lower intention to socially distance [OR = .468], had lower control over others’ distancing [OR = .829], had a doctoral degree compared to a master’s degree [OR = .332], a professional qualification [OR = .307], a bachelor’s degree [OR = .361] or work-related qualification [OR = .174], voted for the UK Government compared to not voting for the Government [OR = .461], perceived higher normative pressure from neighbours [OR = 1.121] and had greater support from friends [OR = 1.465].

**Conclusions:**

Non-adherence to all SD rules had a stronger association with vulnerability to COVID-19 and control over SD, whereas intentional non-adherence had a stronger association with intention and anti-social psychological factors. It is recommended that people living in high-risk environments, such as those living in houses of multiple occupancy, should be specially supported when asked to stay at home, and public health messaging should emphasise shared responsibility and public consciousness.

**Supplementary Information:**

The online version contains supplementary material available at 10.1186/s12889-021-10379-7.

## Background

On March 23, 2020, the government of the United Kingdom (UK) introduced unprecedented public health measures to slow the spread of COVID-19, a virus which transfers rapidly from human to human [1]. A policy of suppression (commonly known as ‘lockdown’ measures) aimed to halt the spread of the epidemic and the number of cases [2] and included social distancing (SD) behaviours. The UK guidelines required the public to stay at home and only leave to exercise once a day, to travel to and from work when work from home was not possible, to shop for essential items, and to fulfil any medical or care needs. When leaving their home for permitted reasons, people were asked to minimise the time spent outdoors and to keep a minimum distance of two metres away from others outside their household. In addition, a shielding policy for extremely vulnerable people as well as self-isolation for 7 days (for those who develop symptoms) and quarantine for 14 days (for those without symptoms but in contact with someone who did) were also part of the SD measures introduced [3]. The Police were given powers to enforce this policy dispersing gatherings in parks, ensuring SD in crowed shops or break up a house party, including powers to issue people with fines for flouting the rules [4].

Although there is evidence that COVID-19 droplets travel beyond two metres [5, 6], SD measures have proven effective in the UK. For example, a second UK lockdown was brought into effect on 5 November 2020 in the UK and, after a 1 week lag, coincided with a significant drop in cases from a peak of 33,470 cases on 12 November 2020 to 11,299 cases on 24 November 2020, as reported by Public Health England [7]. In addition, a recent study by Brauner et al. [8] has modelled the effectiveness of non-pharmaceutical interventions in 41 countries concluding that reducing physical contact such as closing schools and universities, limiting gatherings to 10 people or less, and closing face-to-face businesses “each reduced transmission considerably” ( [8] , p. 1). This evidence suggests that, until a vaccine provides herd immunity, SD and other non-pharmacological interventions are the most effective tools in limiting infections of COVID-19.

A critical aspect of non-pharmaceutical interventions, such as SD, is that they primarily rely on population behaviour change, which requires acceptance and more importantly, adherence to the measures. In the UK, there has been a perceived sense of people’s adherence to SD rules, as observed in the Government’s declarations that “The overwhelming majority of the British public have complied with the regulations and guidance” [9] and the Independent Sage Group’s observation of “the impressively sustained high levels of adherence to lockdown the public has achieved” [10]. Yet early data in May 2020 started to show an increase in peoples’ mobility even before the Government relaxation of lockdown measures on the 13th of May: according to Google Mobility report, visits to parks exceeded pre-lockdown rates [11] and data from the Department of Transport showed the use of cars was increasing to a 50% of that observed before SD rules by the 18th of May [12].

Maintaining SD and movement restrictions to reduce the spread of a virus have been previously used by many countries to tackle other respiratory pandemics, and there is a body of evidence assessing population responses to various preventative measures. It is well established that several demographic, psychological and social factors are associated with engagement of SD behaviours during a pandemic, even when findings suggests caution in generalisations. Women are more likely to avoid crowds and use of public transport than men [13, 14]; older age is often associated to engagement with SD behaviours [13–15], though other studies have found no conclusive evidence [16, 17]. Black, Asian and Minority Ethnic (BAME) populations in the UK have been found to be at greater risk of dying from COVID-19 compared to the white population [18], and evidence suggest this is multi-factorial (material deprivation, increased exposure to risk and structural racism amongst others [19], yet findings from previous pandemics and COVID-19 suggest that this is not due to poorer adherence to SD measures [20, 21]. Higher socio-economic status and higher educational attainment are often related to engagement in SD behaviours [14–16, 22], although both low income and having no qualifications have also been associated with greater adherence to SD behaviours in a UK study [20].

In terms of psychological factors, perceived susceptibility to becoming infected by the virus is usually considered to increase the likelihood of compliance with SD behaviours [17, 23–25], though some studies did not find consistent associations [13, 26]. As perceived severity increases (i.e. an individual’s perceived fatality or potential health seriousness if infected), so does engagement with preventative behaviours [14, 17, 20, 27]. Insight into this relationship comes from Teasdale and Yardley [28], who found that if risk is perceived to be low, stay at home messages were perceived to be extreme and inappropriate. Also, engaging in a preventative behaviour is more likely if an individual believes that it will be effective, if they do not perceive barriers to engagement and if they perceive themselves to be competent in successfully performing the preventative behaviour [20, 25, 26].

Greater knowledge about a disease, in particular about symptoms and SD measures, is positively associated with adherence [16, 29, 30], although some studies [15, 23, 31] have shown that knowledge alone is not sufficient and other factors such as perceived susceptibility, perceived behavioural control and intention may play a more determinant role. In addition, a low sense of social responsibility and social consciousness coupled with self-interest values, such as individuals being more concerned about the risk to themselves rather than the risk they would pose to others, have also been associated with non-adherence to SD measures [27, 32].

More recently, political factors have been associated with SD behaviours during the COVID-19 pandemic as shown in the United States where it became a bipartisan issue, whereby supporters of the Republican government were found to engage in less SD behaviours than the Democrats due to a lower perception of risk associated with the virus [33–35].

Primary research and reviews on previous pandemics alongside emerging research in COVID-19 collectively have evidenced a range of variables and often contested behavioural and demographic associations in terms of adherence to SD measures, thus indicating that responses to global pandemics are context-specific. Yet studies on adherence remain reduced to discrete variables and often embedded in research analysing preventative hygiene-related behaviours, such as hand washing, use of masks, covering cough and sneeze. In this sense, to the best of our knowledge, there is no literature that has sought to understand the interplay of demographic, housing, health, political, psychological and social factors in influencing people’s adherence to SD rules during the COVID-19 pandemic, a gap this study aims to address. In modelling the explanatory variables previously found to be significant predictors of adherence, we drew on different constructs within the behavioural sciences, such as the Protection Motivation Theory (PMT) [36], the Theory of Planned Behaviour (TPB) [37] and the Socio-Ecological Model (SEM) [38]. Whilst PMT and TPB informed the exploration of psychological variables, this study also sought to contextualise intrapersonal level factors by analysing socio-environmental factors, as proposed by SEM, that could be associated with behavioural adherence.

In addition, this study uniquely distinguished between non-adherence to all SD rules (intentional and unintentional), and intentional non-adherence to SD rules: Unintentional non-adherence indicates inability to follow the rules due to practical issues (perceived behavioural control e.g. keeping two metres apart at all times, or not controlling other’s distance), and to individual’s capacity (self-efficacy e.g. misunderstanding of the rules, or even forgetting the rules); whilst intentional non-adherence refers to individuals consciously and deliberately not following SD rules or following them partially, due to beliefs, preferences or priorities [39]. Due to the different psychological and social-environmental processes involved in intentional and unintentional non-adherence, it was hypothesised that significant differences will be observed between the two models, and different interventions would be needed to tackle them.

By using a convenience sample of North London residents, the aim of this study was to analyse the key demographic and psycho-social factors associated with non-adherence to SD measures. The research questions the study explored were: 1) What are the demographic, housing, health, political, psychological and social factors associated with non-adherence of all SD rules by North London residents?; 2) What are the demographic, housing, health, political, psychological and social factors associated with intentional non-adherence of SD rules by North London residents?

## Methods

### Design

The study was designed as a cross-sectional survey to be administered via convenience sampling among adults in North London. To be included in the study, participants were required to be aged ≥ 18 years and a resident in the London boroughs of Islington, Haringey, Camden, Hackney, Barnet or Enfield. The total population of the qualifying boroughs is 1,777,666 [40]. In specifying a 99% confidence level and 5% margin of error, the minimum sample size required for this population is 663 [41]. Data were collected via a digital questionnaire using JISC’s online surveys software, which meets information security standards and is GDPR compliant [42]. The questionnaire went live on 1st of May, 2020, and was closed on 31st of May, 2020, at midnight, coincidentally at the point when lockdown was eased for the second time. An incentive of a random prize draw to win one of four £100 vouchers for the Aldi supermarket was used to encourage questionnaire completion. The study and the questionnaire were promoted via the authors’ university website, local newspapers and social media.

### Instrument

To collect the study data, researchers developed a 15-min self-report questionnaire that covered seven groups of factors using previous scientific literature and scales as well as self-developed items and scales. Sample items of variables can be viewed in Table 1.

**Table 1:** Sample items of research variables

Variables	Sample Items
**Social Distancing**	
Non-adherence	In the past 2 weeks, how many times have you gone out for groceries and come within two metres (approx. 3 steps) of someone (e.g. cashier, other shoppers) you don’t live with?
Intentional non-adherence (unpermitted meeting of others)	In the past 2 weeks, how many times have you broken social distancing rules to meet friends that don’t live with you?
Intentional non-adherence (unpermitted leaving of the house)	In the past 2 weeks, how many times have you gone out for reasons other than to work, to buy groceries, for medical reasons (e.g. to collect a prescription) to enjoy parks or public spaces or to exercise?
**Health Factors**	
Perceived Susceptibility	There is a good chance that I will get coronavirus (COVID-19)
**Political Factors**	
Trust in Government	I trust the UK Government in their response to COVID-19
**Psychological Factors**	
COVID-19 and Social Distancing Knowledge	Wearing latex gloves is more effective than hand washing at protecting against coronavirus (COVID-19)
Social Responsibility	Before I act, I think about how my actions might have a negative effect on others
Self-Interest	I do what I want, regardless of what others want me to do
Intention to Socially Distance	I will only leave my home for permitted reasons for as long as the lockdown measures are in place
Control over Leaving the House	During lockdown, I do not need to leave my home if I don’t want to
Control over Others’ Distancing	When I go out for permitted reasons, I cannot stop others from coming within two metres of me^a^
Control over Responsibilities	I have responsibilities (e.g., work, childcare) for which I cannot avoid coming into contact with others that I do not live with^a^
Family Normative Pressure	My family support staying at home and social distancing
Friends Normative Pressure	My friends are keen to meet up in person, despite the lockdown^a^
Neighbours Normative Pressure	I see my neighbours keeping social distancing rules when they are out in my street
**Social Factors**	
Support from a Special Person	During lockdown, there is a special person who is around when I am in need
Support from Family	During lockdown, my family really tries to help me
Support from Friends	During lockdown, I can talk about my problems with my friends

^a^Reversed items

#### SD behaviours

SD behaviours were measured via six items, which asked participants to recall SD behaviours from the previous 2 weeks. The first three items captured how many times participants had gone out for permitted reasons (i.e., for grocery shopping, medication, exercise or work) and not been able to maintain SD (i.e., they came within two metres of someone not lived with). The next two items captured how many times participants broke SD rules to meet up with others (i.e., extended family or friends). The final item captured how many times participants went out for unpermitted reasons. To create the outcome variable of non-adherence to all SD rules, all violations were summed to create a total violations variable, which was then re-coded to create a binary variable of adherence (coded 0) and non-adherence (coded 1) to all SD rules. To create the outcome variable of intentional non-adherence to SD rules, violations from the final three items, which covered going out for unpermitted reasons and breaking SD rules to meet up with friends and family, were summed to create a total intentional violations variable, which was then re-coded to create a binary variable of intentional adherence (coded 0) and non-adherence (coded 1).

#### Demographic factors

Demographic data was collected about gender, age, ethnicity, whether or not English was the participant’s first language, religion, highest qualification obtained, employment status, key worker status, and deprivation. Item wording and categories were taken directly from the England Census Rehearsal Household Questionnaire [43] where possible. Furthermore, participants were asked whether they were a key worker, as defined by the UK Government, in terms of whether their work is critical to the COVID-19 response. Also, participants were asked to input their post code, which was then input to the English indices of deprivation tool [44], which returned deprivation data. From this, the index of multiple deprivation decile for each participant’s postcode was recorded, a score from one to ten, with one representing most deprived and ten representing least deprived, which was treated as continuous data in the analysis. London Borough was also recorded from the tool, which was subsequently used to verify if a participant was a resident from a target London Borough and their data excluded if not, although this was not used as a variable in the analysis.

#### Housing factors

Participants were asked to identify their housing situation, in terms of whether they lived in their own home, a rented home, or a rented room in a house of multiple occupancy, how many people they lived with, and whether they lived with someone vulnerable to COVID-19, such as someone over 70 years old or with a health condition that made them more vulnerable.

#### Health factors

Participants were asked whether, as defined by the UK Government, they had a medical condition which made them more vulnerable to COVID-19 and whether they had experienced COVID-19 symptoms. Perceived susceptibility (PMT) was measured via a single item, adjusted from a single item measuring perceived susceptibility to cancer [45].

#### Political factors

Participants were asked which political party they voted for in the 2019 General Election with response options for all major political parties. Due to the low number of responses for parties other than Labour or the Conservatives this data was recoded as not voting for the Government (i.e., not voting Conservative) or voting for the Government (i.e. voting Conservative). Trust in the Government (3 items, α = .888) was self-developed and covered trust in the response to COVID-19 and trust in the scientific advice. During data collection, lockdown restrictions were relaxed by the Government. Hence, to control for this and measure any effect of this change, the dates of participants’ submission of response were coded as total lockdown if submitted by the final day of total lockdown on Tuesday 12th May, 2020. Given that participants were asked to recall SD behaviours over 2 weeks, responses up to Tuesday 26 May were coded as overlap of total and first relaxation. Responses from Wednesday 27th May recalled behaviours that were specific to the first relaxation phase and were coded as such. Further relaxation of lockdown rules occurred on 1st June, 2020, but as planned, data collection ended on 31st of May.

#### Psychological factors

COVID-19 and SD knowledge were measured via a self-developed quiz. Nine statements were developed from the World Health Organization’s COVID-19 myth busters web portal and from the UK Government’s guidance on SD rules, some trues and some false, against which participants had to select true, false or do not know. Single items for self-interest and social responsibility were adjusted from Oosterhoff and Palmer [27]. Using the TPB [37] as a guide, a scale was self-developed for SD behavioural intention (3 items, α = .854), three items covering perceived behavioural control and three items covering normative pressure from family, friends and neighbours. Control items (3 items, α = .354) and normative pressure items (3 items, α = .254) were modelled separately due to Cronbach’s alpha scores being below the threshold of 0.7 for sufficient internal consistency [46]. Although these items were originally grouped in line with the TPB for constructs of perceived behavioural control and normative pressures, as revealed by the analysis, the items are indirect measures and it is expected that their internal consistency will be insufficient. In other words, feeling normative pressure from family is independent of feeling normative pressure from friends or neighbours. Therefore, it is recommended to model these beliefs separately [47].

#### Social factors

Informed by the SEM, participants were asked to report if during the lockdown they were receiving financial and community support if needed. Social support was measured using the multidimensional scale of perceived social support [48], with items clarified to refer to the lockdown period. Sub-scales for support from a special person (3 items, α = .939), family (3 items, α = .937) and friends (3 items, α = .94) were used.

## Statistical analysis

To measure the associations between explanatory variables (i.e., demographic, housing, health, political, psychological and social factors) and outcome variables (i.e., non-adherence of *all* SD rules and intentional non-adherence of SD rules) univariate, multivariate and mapping analysis were undertaken. For univariate analysis, Pearson’s chi-square tests were ran to identify statistically significant univariate associations between categorical explanatory variables and the binary outcome variables. Independent sample t-tests were ran to detect statistically significant univariate differences between the means of continuous explanatory variables for participants that did not adhere to all SD and those that did. A logistic regression model was run to measure the multivariate associations between explanatory variables and the two binary outcome variables, from which odds ratios for each explanatory variable with the corresponding 95% confidence interval (CI) and *P*–value were presented. Where there were significant univariate analysis associations that were not found in multivariate analysis further mapping analysis was conducted to determine the significant explanatory variable that better accounted for variation in the outcome variable in the multivariate analysis, providing a more vivid understanding of non-adherence to SD rules. To identify associations between two categorical variables Pearson’s chi-square tests were used, between categorical and continuous variables independent sample t-tests were used where there were two categories and ANOVA where there were more than two categories and between two ordinal variables Spearman correlations were used.

## Results

### Participants

There were a total of 701 responses to the study’s questionnaire. Of these, 20 responses came from locations other than the specified North London boroughs and so were removed from the dataset, leaving a sample of 681 participants, the characteristics of who are reported in Table 2. The sample was highly skewed to females, with 82.8% of respondents being female (564 vs. 111 males). This over-representation reflects the well-established trend that women are more likely to participate in surveys than men [49–52], which has been explained in terms of gender differences, such as greater empathy and emotional closeness in females, which are associated with greater survey participation [52, 53]. The average age was 42.43 years old. A minority of 14.4% of participants came from BAME populations (98 vs. 583 White) and 14.5% did not have English as their first language. Despite the recruitment strategy included deliberately targeting ethnic minorities through local communities and neighbourhoods’ social media pages, the sample is disproportionate to the 40.2% of the broader London population who come from BAME groups [54]. This under-representation reflects the well-established trend that ethnic minorities are less likely to participate in health surveys than ethnic majorities [55, 56], however the reasons for this are complex including linguistic and educational limitations [56] as well as mistrust of research and entrenched structural inequalities [57]. The majority of participants (61.7%; 420) had no religion and the majority of participants had either a bachelor’s (34.7%; 236) or master’s (29.1%; 198) degree. The most common employment status was working as an employee from home (39.4%; 268) and 22.5% (153) of participants were key workers. Twelve per cent (82) of participants lived in a rented room in a house of multiple occupancy, the average number of people each participant lived with was 2.57 and 15.3% (104) of participants lived with a person of vulnerable health status. Fifteen per cent (102) of participants were vulnerable to COVID-19 and 30.7% (209) had previously had COVID-19 symptoms. The average level of perceived susceptibility was 4.4 on a scale of a 1–7. The vast majority (91%; 620) of participants did not vote for the Government and the sample had relatively low level of trust (2.96 on a scale of 1–7) in them. Survey responses were distributed evenly across the three categories of lockdown phase (259 responses during the first total lockdown, 255 responses during a period of overlap between the first total lockdown and the subsequent first relaxation of rules and 167 responses after the first relaxation of rules). Knowledge about COVID-19 and SD rules was, on average, high (7.03 out of 9). Social responsibility was high (6.19 out of 7) and self-interest was low (1.81). Intention to socially distance (5.95 out of 7), control over leaving the house (5.34), control over others’ distancing (5.48) were all high, but control over responsibilities was relatively low (2.81). Normative pressure to socially distance was highest from family (6.29 out of 7), followed by friends (5.52) and neighbours (4.69). Twenty percent (136) of participants were not getting the financial support they needed and 12% (82) were not getting the community support they needed.

**Table 2:** Characteristics of sample

Explanatory Variables	n	%	Mean	S.D.	Min.	Max.
**Demographic Factors**						
Gender						
Female	564	82.8				
Male	111	16.3				
Other	6	0.9				
Age			42.43	13.62	19	77
Ethnicity						
White	583	85.6				
BAME	98	14.4				
Language						
English as First Language	582	85.5				
English Not as First Language	99	14.5				
Religion						
No Religion	420	61.7				
Christian	154	22.6				
Buddhist	9	1.3				
Hindu	3	0.4				
Jewish	53	7.8				
Muslim	14	2.1				
Sikh	2	0.3				
Other	26	3.8				
Highest Qualification Obtained						
Doctoral Degree	39	5.7				
Masters Degree	198	29.1				
Professional Qualification	81	11.9				
Bachelors Degree	236	34.7				
Vocational / Work-related Qualification	40	5.9				
A Levels or equivalent	44	6.5				
GCSEs or equivalent	30	4.4				
No Qualifications	13	1.9				
Employment Status						
Long-term sick or disabled	28	4.1				
Retired	56	8.2				
Working as an employee from home	268	39.4				
Self-employed or freelance from home	66	9.7				
Looking after home or family	29	4.3				
Unemployed	36	5.3				
A furloughed employee	64	9.4				
A student	20	2.9				
Working as an employee in my normal place of work (not home)	67	9.8				
Self-employed or freelance in my normal place of work (not home)	16	2.3				
Other	31	4.6				
Key Worker Status						
Not Key Worker	528	77.5				
Key Worker	153	22.5				
Deprivation (1–10)			4.42	2.126	1	10
**Housing Factors**						
Housing Situation						
Live in Own Home	349	51.2				
Live in Rented Home	250	36.7				
Live in Rented Room of Multiple Occupancy House	82	12				
Number of People Living With			2.57	1.368	0	9
Living with a Vulnerable Person						
Living with Person of Vulnerable Health Status	104	15.3				
Not Living with Person of Vulnerable Health Status	577	84.7				
**Health Factors**						
Health						
Vulnerable	102	15				
Not Vulnerable	579	85				
COVID-19 Symptoms						
Not Had	472	69.3				
Had	209	30.7				
Perceived Susceptibility (1–7)			4.74	1.557	1	7
**Political Factors**						
2019 General Election						
Voted for Government	61	9				
Did Not Vote for Government	620	91				
Trust in Government (1–7)			2.96	1.541	1	7
Lockdown Phase						
Total Lockdown	259	38				
Overlap of Total and First Relaxation	255	37.4				
First Relaxation	167	24.5				
**Psychological Factors**						
COVID-19 and Social Distancing Knowledge (out of 9)			7.03	1.055	3	9
Self-Control (1–7)			6.19	1.012	1	7
Self-Interest (1–7)			1.81	1.13	1	7
Intention to Socially Distance (1–7)			5.95	1.16	1.67	7
Control over Leaving the House (1–7)			5.34	1.891	1	7
Control over Others’ Distancing (1–7)			5.48	1.558	1	7
Control over Responsibilities (1–7)			2.81	2.197	1	7
Family Normative Pressure (1–7)			6.29	1.075	1	7
Friends Normative Pressure (1–7)			5.52	1.699	1	7
Neighbours Normative Pressure (1–7)			4.69	1.825	1	7
**Social Factors**						
Financial Support						
Getting Financial Support if Needed	545	80				
Not Getting Financial Support If Needed	136	20				
Community Support						
Getting Community Support if Needed	599	88				
Not Getting Community Support If Needed	82	12				
Support from a Special Person (1–7)			5.52	1.846	1	7
Support from Family (1–7)			5.35	1.665	1	7
Support from Friends (1–7)			5.41	1.44	1	7

### Non-adherence of SD rules

As reported in Table 3, the vast majority of participants (92.8%; 632) did not adhere to all SD rules. Similarly, 90.7% (618) of participants were unable to always maintain two metres distance from others when they went out for permitted reasons indicating significant overlap between non-adherence of all rules and unintentional non-adherence. Slightly less than half (48.6%; 331) of participants intentionally did not adhere to SD rules. The more common intentional violation was unpermitted leaving of the house, which a third (227) of participants did not adhere to. Less frequent were unpermitted meeting of others with 28.8% (196) of participants not adhering to this rule.

**Table 3:** Non-adherence of SD rules

	Adherence (n)	Adherence (%)	Non-Adherence (n)	Non- Adherence (%)
All	49	7.2%	632	92.8%
Unintentional	63	9.3%	618	90.7%
Intentional	350	51.4%	331	48.6%
Unpermitted Leaving of House	454	66.7%	227	33.3%
Unpermitted Meeting of Others	485	71.2%	196	28.8%

### Factors associated with non-adherence of all SD rules

#### Univariate analysis

The distribution of participants who did not adhere to SD rules and those that did across categories of the categorical variables are reported in Table 4. There were statistically significant univariate associations between the following categorical explanatory variables and non-adherence to all SD rules: employment status (χ^2^(10) = 36.986, *p* = .000); housing situation (χ^2^(2) = 7.659, *p* = .022); living with a person of vulnerable health status (χ^2^(1) = 7.218, *p* = .007); and vulnerable health status (χ^2^(2) = 23.48, *p* = .000). There were no statistically significant univariate associations (*p* > .05) between the explanatory variables of gender, ethnicity language, religion, highest qualification obtained, key worker status, COVID-19 symptoms, voting for the Government, lockdown phase, financial support and community support and non-adherence of all SD rules.

**Table 4:** Percentage of participants who did not adhere to all SD rules and those who did by categorical explanatory variable

Explanatory Variables	Adherence (n)	Adherence (%)	Non-Adherence (n)	Non- Adherence (%)
Sample	49	7%	632	93%
**Demographic Factors**				
Gender				
Female	42	7.4%	522	92.6%
Male	5	4.5%	106	95.5%
Other	2	33.3%	4	66.7%
Ethnicity				
White	41	7%	542	93%
BAME	8	8.2%	90	91.8%
Language				
English as First Language	43	7.4%	539	92.6%
English Not as First Language	6	6.1%	93	93.9%
Religion				
No Religion	24	5.7%	396	94.3%
Christian	15	9.7%	139	90.3%
Buddhist	1	11.1%	8	88.9%
Hindu	1	33.3%	2	66.7%
Jewish	4	7.5%	49	92.5%
Muslim	1	7.1%	13	92.9%
Sikh	0	0%	2	100%
Other	3	11.5%	23	88.5%
Highest Qualification Obtained				
Doctoral Degree	1	2.6%	38	97.4%
Masters Degree	11	5.6%	187	94.4%
Professional Qualification	7	8.6%	74	91.4%
Bachelors Degree	22	9.3%	214	90.7%
Vocational / Work-related Qualification	6	15%	34	85%
A Levels or equivalent	0	0%	44	100%
GCSEs or equivalent	1	3.3%	29	96.7%
No Qualifications	1	7.7%	12	92.3%
Employment Status^a^				
Long-term sick or disabled	5	17.9%	23	82.1%
Retired	13	23.2%	43	76.8%
Working as an employee from home	13	4.9%	255	95.1%
Self-employed or freelance from home	5	7.6%	61	92.4%
Looking after home or family	4	13.8%	25	86.2%
Unemployed	1	2.8%	35	97.2%
A furloughed employee	3	4.7%	61	95.3%
A student	1	5%	19	95%
Working as an employee in my normal place of work (not home)	1	1.5%	66	98.5%
Self-employed or freelance in my normal place of work (not home)	0	0%	16	100%
Other	3	9.7%	28	90.3%
Key Worker Status				
Not Key Worker	43	8.1%	485	91.9%
Key Worker	6	3.9%	147	96.1%
**Housing Factors**				
Housing Situation^a^				
Live in Own Home	34	9.7%	315	90.3%
Live in Rented Home	13	5.2%	237	94.8%
Live in Rented Room of Multiple Occupancy House	2	2.4%	80	97.6%
Living with a Vulnerable Person^a^				
Living with Person of Vulnerable Health Status	14	13.5%	90	86.5%
Not Living with Person of Vulnerable Health Status	35	6.1%	542	93.9%
**Health Factors**				
Vulnerable Health^a^				
Vulnerable	19	18.6%	83	81.4%
Not Vulnerable	30	5.2%	549	94.8%
COVID-19 Symptoms				
Not Had	37	7.8%	435	92.2%
Had	12	5.7%	197	94.3%
**Political Factors**				
2019 General Election				
Voted for Government	6	9.8%	55	90.2%
Did Not Vote for Government	43	6.9%	577	93.1%
Lockdown Phase				
Total Lockdown	17	6.6%	242	93.4%
Overlap of Total and First Relaxation	14	5.5%	241	94.5%
First Relaxation	18	10.8%	149	89.2%
**Social Factors**				
Financial Support				
Getting Financial Support if Needed	38	7%	507	93%
Not Getting Financial Support If Needed	11	8.1%	125	91.9%
Community Support				
Getting Community Support if Needed	43	7.2%	556	92.8%
Not Getting Community Support If Needed	6	7.3%	76	92.7%

^a^Statistically significant association

The differences in means of continuous explanatory variables between participants who did not adhere to all SD rules and those that did are reported in Table 5. Participants that did not adhere to all SD rules had statistically significantly: lower age (41.69 ± 13.313) compared to those who adhered (52.05 ± 14.016), t (679) = 5.226, *p* = .000; higher perception of susceptibility (4.79 ± 1.543) compared to those who adhered (4 ± 1.586), t (679) = − 3.461, *p* = .001; lower intention to socially distance (5.91 ± 1.168) compared to those who adhered (6.42 ± .924), t (679) = 2.943, *p* = .003; lower control over leaving the house (5.28 ± 1.923) compared to those who adhered (6.08 ± 1.187), t (679) = 2.863, *p* = .004; lower control over others’ distancing to them (2.45 ± 1.516) compared to those who adhered (3.41 ± 1.813), t (679) = 4.208, *p* = .000; lower control over their responsibilities (5.1 ± 2.228) compared to those who adhered (6.35 ± 1.285), t (679) = 3.852, *p* = .000; and lower perception of normative pressure from friends (5.47 ± 1.718) compared to those who adhered (6.24 ± 1.234), t (679) = 3.101, *p* = .002. There were no statistically significant differences (*p* > .05) in deprivation, number of people living with, trust in government, knowledge, social responsibility, self-interest, normative pressure from family, normative pressure from neighbours, support from a special person, support from family and support from friends.

**Table 5:** Comparison of means of continuous explanatory variables between participants who did not adhere to all SD rules and those that did

Explanatory Variables	Adherence (Mean)	Adherence (S.D.)	Non-Adherence (Mean)	Non- Adherence (S.D.)
**Demographic Factors**				
Age^a^	52.04	14.016	41.69	13.313
Deprivation	4.84	2.418	4.39	2.1
**Housing Factor**				
Number of People Living With	2.43	1.242	2.59	1.378
**Health Factor**				
Perceived Susceptibility^a^	4	1.568	4.79	1.543
**Political Factor**				
Trust in Government	3.05	1.626	2.96	1.535
**Psychological Factors**				
COVID-19 and Social Distancing Knowledge	6.98	.946	7.03	1.064
Social Responsibility	6.37	.859	6.18	1.022
Self-Interest	1.8	1.258	1.81	1.12
Intention to Socially Distance^a^	6.42	.924	5.91	1.168
Control over Leaving the House^a^	6.08	1.187	5.28	1.923
Control over Others’ Distancing^a^	3.41	1.813	2.45	1.516
Control over Responsibilities^a^	6.35	1.284	5.1	2.228
Normative Pressure from Family	6.53	.793	6.27	1.092
Normative Pressure from Friends^a^	6.24	1.234	5.47	1.718
Normative Pressure from Neighbours	5.12	1.728	4.66	1.83
**Social Factors**				
Support from a Special Person	5.63	1.811	5.51	1.85
Support from Family	5.44	1.691	5.34	1.664
Support from Friends	5.37	1.32	5.42	1.45

^a^Statistically significant difference

#### Multivariate analysis

The logistic regression model was statistically significant, χ^2^(57) = 125.288, *p* = .000, explained 41.6% (Nagelkerke *R*^*2*^) of the variance in non-adherence to all SD rules and correctly classified 93.4% of cases. The results of the logistic regression are reported in Table 6. When holding other factors constant, the odds of not adhering to all SD rules are 73.9% lower if reporting after lockdown rules had been relaxed for the first time than if reporting during total lockdown. When holding other factors constant, the odds of not adhering to all SD rules are 350.6% higher if the person is not vulnerable than if vulnerable. An additional level of agreement on a 7-point Likert scale about perception of control over others’ distancing decreases the odds of not adhering to all SD rules by 27.6%. An additional level of agreement on a 7-point Likert scale about perception of control over responsibilities, for which coming into contact with others outside the household is unavoidable, decreases the odds of not adhering to all SD rules by 35.8%.

**Table 6:** Results of logistic regression, with binary outcome variable of non-adherence or adherence to all social distancing rules

Explanatory Variables	Exp (B)	95% Wald Confidence Interval for Exp (B)		Sig.
		Lower	Upper	
Constant	24,481,821.0			.996
**Demographic Factors**				
Gender				.047
Female				
Male	1.305	.388	4.389	.667
Other^b^	.047	.004	.592	.018
Age	.977	.933	1.023	.322
Ethnicity				
White				
BAME	1.19	.37	3.832	.77
Language				
English as First Language				
English Not as First Language	.613	.182	2.068	.43
Religion				.488
No Religion				
Christian	.504	.203	1.252	.14
Buddhist	6.469	.208	201.301	.287
Hindu	.099	.003	3.111	.188
Jewish	1.641	.298	9.043	.569
Muslim	1.171	.07	19.652	.913
Sikh	5.882E+ 9	.000		.999
Other	.414	.071	2.407	.326
Highest Qualification Obtained				.306
Doctoral Degree				
Masters Degree	.353	.029	4.343	.416
Professional Qualification	.21	.016	2.811	.238
Bachelors Degree	.166	.014	2.028	.16
Vocational / Work-related Qualification	.133	.008	2.121	.153
A Levels or equivalent	28,639,096.0	.000		.997
GCSEs or equivalent	1.272	.042	38.118	.89
No Qualifications	2.406	.041	141.664	.673
Employment Status				.586
Long-term sick or disabled				
Retired	.787	.136	4.567	.79
Working as an employee from home	3.579	.682	18.774	.132
Self-employed or freelance from home	2.24	.36	13.947	.387
Looking after home or family	.721	.075	6.904	.777
Unemployed	3.303	.23	47.51	.38
A furloughed employee	3.573	.451	28.315	.228
A student	1.313	.071	24.42	.855
Working as an employee in my normal place of work (not home)	2.605	.136	49.896	.525
Self-employed or freelance in my normal place of work (not home)	121,440,088	.000		.998
Other	3.414	.363	32.075	.283
Key Worker Status				
Not Key Worker				
Key Worker	.511	.154	1.694	.272
Deprivation	1.016	.841	1.227	.873
**Housing Factors**				
Housing Situation				.909
Live in Own Home				
Live in Rented Home	1.054	.374	2.971	.920
Live in Rented Room of Multiple Occupancy House	1.553	.214	11.276	.664
Number of People Living With	.849	.619	1.166	.312
Living With a Vulnerable Person				
Living with Person of Vulnerable Health Status				
Not Living with Person of Vulnerable Health Status	.998	.385	2.586	.997
**Health Factors**				
Vulnerable Health^a^				
Vulnerable				
Not Vulnerable^a^	4.506	1.799	11.285	.001
COVID-19 Symptoms				
Not Had				
Had	.87	.341	2.215	.77
Perceived Susceptibility	1.102	.823	1.477	.514
**Political Factors**				
2019 General Election				
Voted for Government				
Did Not Vote for Government	1.522	.361	6.41	.567
Trust in Government	1.005	.76	1.328	.974
Lockdown Phase^a^				.009
Total Lockdown				
Overlap of Total and First Relaxation	1.204	.465	3.114	.702
First Relaxation^a^	.261	.096	.711	.009
**Psychological Factors**				
COVID-19 and Social Distancing Knowledge	1.273	.864	1.877	.223
Social Responsibility	.966	.63	1.482	.875
Self-Interest	.985	.707	1.372	.927
Intention to Socially Distance	.688	.433	1.094	.114
Control over Leaving the House	.821	.634	1.063	.135
Control over Others’ Distancing^a^	.724	.573	.916	.007
Control over Responsibilities^a^	.642	.474	.869	.004
Normative Pressure from Family	.931	.577	1.5	.768
Normative Pressure from Friends	.739	.526	1.037	.08
Normative Pressure from Neighbours	1.062	.824	1.369	.643
**Social Factors**				
Financial Support				
Getting Financial Support if Needed				
Not Getting Financial Support If Needed	.731	.241	2.22	.58
Community Support				
Getting Community Support if Needed				
Not Getting Community Support If Needed	1.166	.27	5.039	.837
Support from a Special Person	.912	.674	1.234	.551
Support from Family	.903	.596	1.366	.628
Support from Friends	1.18	.758	1.838	.464

^a^Significant predictors of non-adherence of all social distancing rules

^b^Significant association found, but below threshold of 10 units per variable (68)

There were no statistically significant multivariate associations (*p* > .05) between the explanatory variables of gender, age, ethnicity, language, religion, highest qualification obtained, employment status, key worker status, deprivation, housing situation, number of people living with, living with a vulnerable person, COVID-19 symptoms, perceived susceptibility, voting for the Government, trust in the Government, knowledge, social responsibility, self-interest, control over leaving the house, normative pressure from family, normative pressure from friends, normative pressure from neighbours, financial support, community support, support from a special person and support from family and the outcome variable of non-adherence of all SD rules.

#### Mapping analysis

In the multivariate analysis, vulnerable health better accounted for variance in non-adherence of all SD rules than age (participants that were not vulnerable were of a statistically significantly lower age (41.49 ± 13.321) compared to participants that were vulnerable (47.75 ± 14.083), *t* (679) = 4.33, *p* = .000), employment status (there was a statistically significant relationship between employment status and vulnerable health (χ^2^(10) = 46.825, *p* = .000) and 50% of long-term sick or disabled were also being classified as vulnerable), living with a vulnerable person (significantly associated with vulnerable health (χ^2^(1) = 11.628, *p* = .001) whereby twice as many vulnerable participants lived with another vulnerable person than non-vulnerable participants) and intention to socially distance (participants that were vulnerable had greater intention to socially distance (6.2908 ± .95403) compared to participants that were not vulnerable (5.8877 ± 1.18212), *t* (160.967) = 3.786, *p* = .000).

In the multivariate analysis, control over others’ distancing better accounted for variance in non-adherence of all SD rules than housing situation (categories differed significantly in terms of control over others’ distancing, *F* (2,678) = 5.313, *p* = .005)) and perceived susceptibility (there was a weak negative correlation between perceived susceptibility and sense of control over others’ distancing, which was statistically significant, *r*_*s*_ (679) = −.231, *p* = .000).

In the multivariate analysis, control over responsibilities better accounted for variance in non-adherence of all SD rules than control over leaving the house (there was a weak, positive correlation between sense of control over leaving the house and sense of control over responsibilities, which was statistically significant, *r*_*s*_ (679) = .263, *p* = .000)) and normative pressure from friends (there was a weak, positive correlation between normative pressure from friends and control over responsibilities, which was statistically significant, *r*_*s*_ (679) = .186, *p* = .000)).

### Factors associated with intentional non-adherence of SD rules

#### Univariate analysis

The distribution of participants who intentionally did not adhere to SD rules and those that did not intentionally break SD rules across categories of the categorical variables are reported in Table 7. There were statistically significant univariate associations between the following categorical variables and intentional non-adherence to SD rules: employment status (χ^2^(10) = 20.248, *p* = .027), housing situation (χ^2^(2) = 7.542, *p* = .023) and vulnerable health status (χ^2^(1) = 6.187, *p* = .013). There were no statistically significant univariate associations (*p* > .05) between the categorical explanatory variables of gender, ethnicity, language, religion, highest qualification obtained, key worker status, living with a vulnerable person, COVID-19 symptoms, voting for the Government, lockdown phase, financial support and community support and intentional non-adherence of SD rules.

**Table 7:** Percentage of participants who intentionally did not adhere to all SD rules and those that did not intentionally break SD rules by categorical explanatory variable

Explanatory Variables	Intentional Adherence (n)	Intentional Adherence (%)	Intentional Non-Adherence (n)	Intentional Non- Adherence (%)
Sample	350	51.4%	331	48.6%
**Demographic Factors**				
Gender				
Female	292	51.8%	272	48.2%
Male	55	49.5%	56	50.5%
Other	3	50%	3	50%
Ethnicity				
White	302	51.8%	281	48.2%
BAME	48	49%	50	51%
Language				
English as First Language	307	52.7%	275	47.3%
English Not as First Language	43	43.4%	56	56.6%
Religion				
No Religion	210	50%	210	50%
Christian	79	51.3%	75	48.7%
Buddhist	5	55.6%	4	44.4%
Hindu	2	66.7%	1	33.3%
Jewish	27	50.9%	26	49.1%
Muslim	7	50%	7	50%
Sikh	2	100%	0	0%
Other	18	69.2%	8	30.8%
Highest Qualification Obtained				
Doctoral Degree	14	35.9%	25	64.1%
Masters Degree	105	53%	93	47%
Professional Qualification	46	56.8%	35	43.2%
Bachelors Degree	117	49.6%	119	50.4%
Vocational / Work-related Qualification	26	65%	14	35%
A Levels or equivalent	23	52.3%	21	47.7%
GCSEs or equivalent	11	36.7%	19	63.3%
No Qualifications	8	61.5%	5	38.5%
Employment Status^a^				
Long-term sick or disabled	19	67.9%	9	32.1%
Retired	30	53.6%	26	46.4%
Working as an employee from home	146	54.5%	122	45.5%
Self-employed or freelance from home	36	54.5%	30	45.4%
Looking after home or family	12	41.4%	17	58.6%
Unemployed	17	47.2%	19	52.8%
A furloughed employee	26	40.6%	38	59.4%
A student	6	30%	14	70%
Working as an employee in my normal place of work (not home)	37	55.2%	30	44.8%
Self-employed or freelance in my normal place of work (not home)	3	18.8%	13	81.3%
Other	18	58.1%	13	41.9%
Key Worker Status				
Not Key Worker	276	52.3%	252	47.7%
Key Worker	74	48.4%	79	51.6%
**Housing Factors**				
Housing Situation^a^				
Live in Own Home	181	51.9%	168	48.1%
Live in Rented Home	138	55.2%	112	44.8%
Live in Rented Room of Multiple Occupancy House	31	37.8%	51	62.2%
Living with Vulnerable Person				
Living with Person of Vulnerable Health Status	62	59.6%	42	40.4%
Not Living with Person of Vulnerable Health Status	288	49.9%	289	50.1%
**Health Factors**				
Health^a^				
Vulnerable	64	62.7%	338	37.3%
Not Vulnerable	286	49.4%	293	50.6%
COVID-19 Symptoms				
Not Had	247	52.3%	225	47.7%
Had	103	49.3%	106	50.7%
**Political Factors**				
2019 General Election				
Voted for Government	26	42.6%	35	57.4%
Did Not Vote for Government	324	52.3%	296	47.7%
Lockdown Phase				
Total Lockdown	137	52.9%	122	47.1%
Overlap of Total and First Relaxation	133	52.2%	122	47.8%
First Relaxation	80	47.9%	87	52.1%
**Social Factors**				
Financial Support				
Getting Financial Support if Needed	287	52.7%	258	47.3%
Not Getting Financial Support If Needed	63	46.3%	73	53.7%
Community Support				
Getting Community Support if Needed	308	51.4%	291	48.6%
Not Getting Community Support If Needed	42	51.2%	40	48.8%

^a^Statistically significant association

The differences in means of continuous explanatory variables between participants who intentionally did not adhere to all SD rules and those that did not intentionally break SD rules are reported in Table 8. Participants that intentionally did not adhere to SD rules had statistically significantly: higher perception of susceptibility (4.87 ± 1.543) compared to those that did (4.61 ± 1.562), *t* (679) = − 2.147, *p* = .032; lower social responsibility (6.1 ± 1.001) compared to participants that did (6.29 ± 1.015), *t* (679) = − 2.445, *p* = .015; higher self-interest (1.93 ± 1.131) compared to participants that did (1.69 ± 1.118), *t* (679) = − 2.705, *p* = .007; lower intention to socially distance (5.49 ± 1.248) compared to those that did (6.38 ± .875), *t* (587.891) = 10.623, *p* = .000; less control over leaving the house (5.16 ± 1.955) compared to those that did (5.51 ± 1.815), *t* (667.724) = 2.883, *p* = .017; less control over others’ distancing to them (2.34 ± 1.457) compared to those that did (2.68 ± 1.632), *t* (676.757) = 4.208, *p* = .004; less control over their responsibilities that would require them to come into contact with others (4.95 ± 2.236) compared to those that did (5.42 ± 2.138), *t* (679) = 2.776, *p* = .006; lower perceptions of normative pressure from family (6.09 ± 1.186) compared to those that did (6.47 ± .923), *t* (623.028) = 4.656, *p* = .000; lower perceptions of normative pressure from friends (5.19 ± 1.772) compared to those that did (5.84 ± 1.568), *t* (658.357) = 5.011, *p* = .000. There were no statistically significant differences (*p* > .05) between participants that intentionally did not adhere to SD rules and those that did in terms of age, deprivation, number of people living with, trust in government, knowledge, normative pressure from neighbours, support from a special person, support from family and support from friends.

**Table 8:** Comparison of means of continuous explanatory variables between participants who intentionally did not adhere to SD rules and those that did not intentionally break SD rules

Explanatory Variables	Adherence (Mean)	Adherence (S.D.)	Non-Adherence (Mean)	Non- Adherence (S.D.)
**Demographic Factors**				
Age	43.31	13.67	41.5	13.525
Deprivation	4.43	2.117	4.42	2.138
**Housing Factor**				
Number of People Living With	2.51	1.317	2.64	1.418
**Health Factor**				
Perceived Susceptibility^a^	4.61	1.562	4.87	1.543
**Political Factor**				
Trust in Government	3.03	1.56	2.89	1.519
**Psychological Factors**				
COVID-19 and Social Distancing Knowledge	7.1	1.002	6.95	1.105
Social Responsibility^a^	6.29	1.015	6.1	1.001
Self-Interest^a^	1.69	1.118	1.93	1.131
Intention to Socially Distance^a^	6.38	.875	5.49	1.248
Control over Leaving the House^a^	5.51	1.815	5.16	1.955
Control over Others’ Distancing^a^	2.68	1.632	2.34	1.457
Control over Responsibilities^a^	5.42	2.138	4.95	2.236
Normative Pressure from Family^a^	6.47	.923	6.09	1.186
Normative Pressure from Friends^a^	5.84	1.568	5.19	1.772
Normative Pressure from Neighbours	4.62	1.902	4.76	1.741
**Social Factors**				
Support from a Special Person	5.52	1.89	5.52	1.801
Support from Family	5.33	1.713	5.37	1.615
Support from Friends	5.32	1.505	5.53	1.36

^a^Statistically significant difference

#### Multivariate analysis

A logistic regression was performed to ascertain the multivariate association between demographic, housing, health, political, psychological and social factors and the likelihood that participants intentionally did not adhere to SD rules. The logistic regression model was statistically significant, χ^2^(57) = 205.963, *p* = .000. The model explained 34.8% (Nagelkerke *R*^*2*^) of the variance in intentional non-adherence of SD rules and correctly classified 72.5% of cases. The results of the logistic regression are reported in Table 9. When holding other factors constant, the odds of intentionally not adhering to SD rules are 66.8% lower if a participant’s highest qualification is a master’s degree, 69.3% lower if a professional qualification, 63.9% lower if a bachelor’s degree and 82.6% lower if a vocational or work-related qualification, than if a doctoral degree. The odds of intentionally not adhering to SD rules are 53.9% lower if not having voted for the government than if having voted for the government. An additional level of agreement on a 7-point Likert scale about intention to socially distance decreases the odds of intentionally not adhering to SD rules by 53.2%. An additional level of agreement on a 7-point Likert scale about perception of control over others’ distancing decreases the odds of intentionally not adhering to SD rules by 17.1%. An additional level of agreement on a 7-point Likert scale about perceiving normative pressure from neighbours increases the odds of intentionally not adhering to SD rules by 12.1%. An additional level of agreement on a 7-point Likert scale about having support from friends increases the odds of intentionally not adhering to SD rules by 46.5%.

**Table 9:** Results of logistic regression, with binary outcome variable of intentional non-adherence or not intentionally breaking SD rules

Explanatory Variables	Exp (B)	95% Wald Confidence Interval for Exp (B)		Sig.
		Lower	Upper	
Constant	51.866			.999
**Demographic Factors**				
Gender				.649
Female				
Male	.795	.475	1.328	.38
Other	.708	.114	4.382	.71
Age	1.001	.98	1.022	.942
Ethnicity				
White				
BAME	1.204	.68	2.132	.523
Language				
English as First Language				
English Not as First Language	1.484	.851	2.587	.164
Religion				.73
No Religion				
Christian	1.164	.725	1.868	.529
Buddhist	.831	.159	4.33	.826
Hindu	1.264	.085	18.815	.865
Jewish	.872	.414	1.838	.719
Muslim	.521	.132	2.050	.351
Sikh	.000	.000		.999
Other	.423	.156	1.141	.089
Highest Qualification Obtained^a^				.02
Doctoral Degree				
Masters Degree^a^	.332	.147	.749	.008
Professional Qualification^a^	.307	.123	.765	.011
Bachelors Degree^a^	.361	.161	.807	.013
Vocational / Work-related Qualification^a^	.174	.057	.531	.002
A Levels or equivalent	.353	.12	1.037	.058
GCSEs or equivalent	1.125	.325	3.891	.853
No Qualifications	.221	.037	1.335	.1
Employment Status				.052
Long-term sick or disabled				
Retired	1.309	.384	4.461	.667
Working as an employee from home	.638	.219	1.854	.409
Self-employed or freelance from home	.693	.215	2.233	.539
Looking after home or family	1.548	.401	5.975	.526
Unemployed	1.007	.286	3.541	.992
A furloughed employee	1.321	.41	4.253	.641
A student	1.02	.223	4.669	.979
Working as an employee in my normal place of work (not home)	.358	.101	1.275	.113
Self-employed or freelance in my normal place of work (not home)	3.184	.551	18.401	.196
Other	.367	.096	1.399	.142
Key Worker Status				
Not Key Worker	1.559	.91	2.672	.106
Key Worker				
Deprivation	1.019	.929	1.118	.684
**Housing Factors**				
Housing Situation				.155
Live in Own Home				
Live in Rented Home	.902	.569	1.431	.662
Live in Rented Room of Multiple Occupancy House	1.737	.845	3.571	.133
Number of People Living With	1.007	.862	1.178	.926
Living With a Vulnerable Person				
Living with Person of Vulnerable Health Status				
Not Living with Person of Vulnerable Health Status	1.26	.723	2.195	.415
**Health Factors**				
Health				
Vulnerable				
Not Vulnerable	1.335	.773	2.308	.3
COVID-19 Symptoms				
Not Had				
Had	1.041	.684	1.583	.852
Perceived Susceptibility	1.077	.939	1.235	.289
**Political Factors**				
2019 General Election				
Voted for Government				
Did Not Vote for Government^a^	.461	.222	.954	.037
Trust in Government	.907	.794	1.036	.149
Lockdown Phase				.444
Total Lockdown				
Overlap of Total and First Relaxation	.769	.501	1.182	.232
First Relaxation	.792	.487	1.289	.348
**Psychological Factors**				
COVID-19 and Social Distancing Knowledge	.871	.726	1.044	.136
Social Responsibility	.96	.79	1.168	.685
Self-Interest	1.07	.891	1.284	.472
Intention to Socially Distance^a^	.468	.381	.575	.000
Control over Leaving the House	.992	.895	1.1	.877
Control over Others’ Distancing^a^	.829	.73	.942	.004
Control over Responsibilities	.91	.819	1.011	.079
Normative Pressure from Family	.872	.716	1.061	.171
Normative Pressure from Friends	.942	.834	1.063	.331
Normative Pressure from Neighbours^a^	1.121	1.004	1.253	.042
**Social Factors**				
Financial Support				
Getting Financial Support if Needed				
Not Getting Financial Support If Needed	1.596	.938	2.714	.085
Community Support				
Getting Community Support if Needed				
Not Getting Community Support If Needed	.764	.396	1.473	.422
Support from a Special Person	.991	.861	1.141	.904
Support from Family	.853	.694	1.05	.133
Support from Friends^a^	1.465	1.152	1.864	.002

^a^Significant predictors of intentional non-adherence of social distancing rules

There were no statistically significant multivariate associations (*p* > .05) between the explanatory variables of gender, age, ethnicity, language, religion, employment status, key worker status, deprivation, housing situation, number of people living with, living with a vulnerable person, vulnerable health, COVID-19 symptoms, perceived susceptibility, trust in the Government, lockdown phase, knowledge, social responsibility, self-interest, control over leaving the house, control over responsibilities, normative pressure from family, normative pressure from friends, financial support, community support, support from a special person and support from family and the outcome variable of intentional non-adherence of SD rules.

#### Mapping analysis

In the multivariate analysis, intention to socially distance better accounted for variance in intentional non-adherence of SD rules than vulnerable health (participants that were vulnerable had greater intention to socially distance (6.2908 ± .95403) compared to participants that were not vulnerable (5.8877 ± 1.18212), *t* (160.967) = 3.786, *p* = .000)), social responsibility (there was a moderate, positive correlation between social responsibility and intention to socially distance, which was statistically significant, *r*_*s*_ (679) = .311, *p* = .000)), self-interest (there was a weak, negative correlation between self-interest and intention to socially distance, which was statistically significant, *r*_*s*_ (679) = −.257, *p* = .000)), control over leaving the house (there was a weak, positive correlation between control over leaving the house and intention to socially distance, which was statistically significant, *r*_*s*_ (679) = .28, *p* = .000)), control over responsibilities (there was a weak, positive correlation between control over responsibilities and intention to socially distance, which was statistically significant, *r*_*s*_ (679) = .152, *p* = .000)), normative pressure from family (there was a moderate, positive correlation between normative pressures from family and intention to socially distance, which was statistically significant (*r*_*s*_ (679) = .39, *p* = .000)) and normative pressure from friends (there was a moderate, positive correlation between normative pressure from family and intention to socially distance, which was statistically significant, *r*_*s*_ (679) = .384, *p* = .000)).

In the multivariate analysis, control over others’ distancing better accounted for variance in intentional non-adherence of SD rules than housing situation (categories differed significantly in terms of control over others’ distancing, *F* (2,678) = 5.313, *p* = .005) and perceived susceptibility (there was a weak, positive correlation between control over others’ distancing and perceived susceptibility, which was statistically significant (*r*_*s*_ (679) = .231, *p* = .000)).

In the multivariate analysis, highest qualification obtained better accounted for variance in intentional non-adherence of SD rules than employment status (there was a statistically significant relationship between employment status and highest qualification obtained, χ^2^(70) = 184.373, *p* = .000)).

## Discussion

Adherence to all SD rules appears highly challenging for the majority of participants in our sample with only 7.2% reporting being able to adhere to all SD rules over a two-week period. The odds of not adhering to all SD rules increased if a participant was not identified as highly vulnerable to COVID-19, had lower control over others’ distancing, had lower control over responsibilities for which coming into contact with others was unavoidable and if SD behaviours were reported after lockdown was first relaxed. Not being vulnerable was associated with a lower intention to socially distance, lower age, not being retired, long-term sick or disabled, and not living with a person of vulnerable health status. Our findings on vulnerability is consistent with a cross-sectional UK study [58], which found that being clinically vulnerable was associated with fewer outings. Lower control over others’ distancing was associated with living in a rented room in a house of multiple occupancy and with higher perceived susceptibility. The former could indicate unintentional non-adherence due to the physical impossibility of keeping distance from others in overcrowded houses and the latter may suggest a sense of inevitability of becoming infected with COVID-19. Lower control over responsibilities, such as work or childcare, for which coming into contact with others is unavoidable was associated with lower control over leaving the house and a lower perception of normative pressure from friends.

Nearly half of participants (48.6%) intentionally broke SD rules. The odds of intentional non-adherence increased if a participant had a lower intention to socially distance, had lower control over others’ distancing, had a doctoral degree, voted for the UK Government, perceived higher normative pressure from neighbours and had greater support from friends. Lower intention to socially distance is associated with not being vulnerable to COVID-19, being less socially responsible, having higher self-interest, lower control over leaving the house, lower control over responsibilities for which coming into contact with others was unavoidable, lower normative pressure from family and lower normative pressure from friends. Lower control over others’ distancing is associated with living in a rented room in a house of multiple occupancy and higher perceived susceptibility. Highest qualification obtained is associated with employment status. Perceived susceptibility and breaking SD rules was also found elsewhere [13, 26] and in a UK study [31], where those who expressed more fear for the disease left the house more often for non-essential activities.

In comparing the univariate and multivariate predictors in the two models, as reported in Table 10, the non-adherence to all rules model is more driven by vulnerability, whereas the intentional non-adherence model is more driven by intention and anti-social psychological factors. Participants who were vulnerable to COVID-19 were 88% less likely to not adhere to all SD rules, which was the most powerful predictor in the model and was associated with four significant factors in the univariate analysis; being older, being retired or long-term sick or disabled, living with someone else who was also vulnerable and a greater intention to socially distance. Lower control over others’ distancing and responsibilities were also significant predictors in the model of non-adherence to all SD rules. Being identified as vulnerable to COVID-19 may lead to greater control over SD as vulnerable individuals received special support, such as priority slots for supermarket deliveries and help from NHS Volunteer Responders to deliver prescriptions, essential items and food.

**Table 10:** Associations between univariate and multivariate predictors and comparison of models

Non-Adherence All Rules		Intentional Non-Adherence	
Univariate Predictor	Multivariate Predictor	Univariate Predictor	Multivariate Predictor
*• Lower Age* *•* Employment Status of Not Retired or Long-term Sick or Disabled *• Not Living With a Person of Vulnerable Health Status* vs. *Living With a Person of Vulnerable Health Status* *•* Lower Intention to Socially Distance	Not Vulnerable vs. Vulnerable Health	*•* Not Vulnerable vs. Vulnerable Health *•* **Lower Social Responsibility** *•* **Higher Self-Interest** *•* Lower Control over Leaving the House *•* Lower Control over Responsibilities *•* **Lower Normative Pressure from Family** *•* Lower Normative Pressure from Friends	Lower Intention to Socially Distance
*•* Living in a Rented Room in a Multiple Occupancy House vs. Owning Own Home *•* Higher Perceived Susceptibility	Lower Control over Others’ Distancing	*•* Living in a Rented Room in a Multiple Occupancy House vs. Owning Own Home *•* Higher Perceived Susceptibility	Lower Control over Others’ Distancing
*•* Lower Control over Leaving the House *•* Higher Normative Pressure from Friends	Lower Control over Responsibilities	*•* Employment Status of Self-employed or Freelance in Normal Place of Work or Student	**Having a Doctoral Degree vs. a Masters, Professional Qualification, Bachelors Degree or Work-related Qualification**
	*First Relaxation Phase* vs. *Total Lockdown*		**Voted for the Government**
			**Higher Normative Pressure from Neighbours**
			**Greater Support from Friends**

Predictors in italics are unique to the all social distancing rules model

Predictors in bold are unique to the intentional non-adherence model

Intentional non-adherence has a stronger association with intention and anti-social psychological factors. For each additional level of intention to socially distance, the odds of intentionally not adhering to SD rules decreased by 53%, which was the strongest predictor of intentional non-adherence and associated with seven significant factors in the univariate analysis, including a weaker sense of social responsibility and a greater sense of self-interest. Intention to socially distance was also associated with constructs from the TPB, notably, normative pressure from family and friends. Counterintuitively, an additional level of support from friends increased the odds of intentionally not adhering to SD rules by 47%, which can be understood as a risk factor for meeting up with others outside the household when coupled with a greater sense of self-interest and lower sense of social responsibility. Other significant variables associated with intentional non-adherence were political orientation, i.e. not voting for the Conservative Government as opposed to voting for a Socialist-led Labour party in particular, decreased the odds of intentionally not adhering to SD rules by 54%; and educational attainment, i.e. PhD holders had the highest rates (64%) of deliberately breaking SD rules compared to other categories of highest qualification achieved. Both resonates with a sense of elitism and self-interest that has been anecdotally evident in high profile cases of individuals in the UK who have intentionally broken SD rules. Overall, the finding on higher degree qualification contrasts with the large body of research that associates greater educational attainment with adherence [16, 22], although of note is an exception from a UK study about the Swine Flu pandemic [20]. Whilst political preference is in line with a study that found British people with more progressive beliefs as observing more SD behaviour [31], another study on political preference provided no association to compliance [59]. The latter is rather a new topic and more research on these relationships is necessary to establish its precise nature before any specific recommendations can be made for improving adherence in these subgroups.

### Policy implications

#### Non-adherence of all rules

Our findings suggest that adherence to all SD rules appears highly challenging for the majority of participants with only 7.2% of participants being able to adhere over a two-week period.

Those that could adhere were more likely to be vulnerable to COVID-19 with greater control over SD behaviours, as vulnerable individuals received special support, such as priority slots for supermarket deliveries and help from NHS Volunteer Responders to deliver prescriptions, essential items and food. There is a range of measures that could both facilitate adherence to SD measures and others that could complement the effect of preventative measures in relation to SD. To facilitate adherence, a support system for people living in houses of multiple occupancy should enable personal protective behaviours through increasing access to online groceries and medication, whilst local authorities working with community organisations could ensure the distribution of food for people living in high-risk environments. For people having low control over responsibilities, in particular those working outside the home, local authorities should ensure protective measures are enforced in workplaces with adequate monitoring and sanctions. In addition, this study found that the odds of not adhering to all SD rules are 73.9% lower if reporting after lockdown rules had been relaxed for the first time than if reporting during total lockdown for which the lack of clarity in messaging and rules may have been a factor. Ensuring clarity of environmental measures, which after the relaxation of rules have often been subject to contradictory messaging, such as the use of face masks, indoor ventilation, crowding levels, venue types, contact time can also reduce the risk of transmission for those coming into close contact with others [60, 61].

#### Intentional non-adherence

Nearly half of participants intentionally broke SD rules, which was associated with ant-social psychological factors such as a lower sense of social responsibility, a higher sense of self-interest and lower normative pressure from family and friends. This highlights the relevance of educational public health messages in communicating pro-social attitude and behaviour change. Recent COVID-19 studies have argued that public messages instilling empathy and altruistic sentiments (i.e. the significance of keeping those most vulnerable safe) can enhance compliance with self-isolation [62, 63]. As the easing of lockdown measures continued but, at the same time, lockdown in local areas is being reinstated, it is important that public health messaging emphasise the significance of shared responsibility and public consciousness to protect those most vulnerable to the disease. In addition, local authorities should take advantage of unprecedented levels of community engagement and participation, observed during the full lockdown, to continue building social trust and sense of belonging in order to improve acceptability and adherence of SD measures.

### Study limitations

Given the observational nature of the study, it is not possible to control the variables so to discern causal relationships. Although a wide range of explanatory variables were modelled and controlled for, there is evidence of omitted variable bias. Specifically, 58.4% of variance in non-adherence to all SD rules and 65.2% of variance in intentional non-adherence was not accounted for in the logistic regression models. That said, the unpredictability of human behaviour [64, 65] and risk-taking [66] is well-established, thus the predictive power of these models is reasonable. Furthermore, without controlled manipulation of the explanatory variables, simultaneity bias is a threat. For example, contrary to the TPB, not intentionally breaking SD rules was associated with weaker normative pressure from neighbours, which can be understood by the theory of downward social comparisons [67] that individuals often compare themselves with others who are performing poorly to enhance their self-esteem, such that the comparisons participants made depended upon their own SD behaviours. In other words, variance was happening at the same time, rather than independently or dependently. Furthermore, this study made use of a non-probability convenience sample, resulting in differences between the sample and the population of interest, such as over-representation of females and under-representation of BAME participants in the sample, as discussed above. That the convenience sample had a local focus on residents in North London means that findings cannot be confidently generalised to the UK.

## Conclusions

Contrary to a perceived sense of people’s adherence to SD, the vast majority of participants did not adhere to all SD rules and nearly half intentionally did not adhere. Given the lack of approved vaccines for COVID-19, at the time of this study, and the threat of a second wave of cases, it is essential to understand the true extent of non-adherence and the factors which predict it, so that policy and interventions can limit the threat. Participants identified as vulnerable to COVID-19 and with greater control over SD were more likely to adhere to all SD rules, suggesting that services which provide greater control over SD, such as delivery of groceries, essential goods and medicines, should be extended to others, in particular those who live in a rented room in a house of multiple occupancy. Other measures to improve adherence include enforcement of protective measures in workplaces and clear messages regarding environmental risk and exposure for those coming into contact with people they do not live with. Intentional non-adherence to SD rules was more associated with psychological factors than non-adherence to all rules, including a lower intention to SD, which was associated with a lower sense of social responsibility, a higher sense of self-interest and lower normative pressure from family and friends. To counter intentional non-adherence it is recommended that public health messages more strongly emphasise the significance of shared responsibility and public consciousness to protect those most vulnerable to the disease.

## Supplementary Information

**Additional file 1: **Digital questionnaire used.

## Data Availability

The datasets generated during and/or analysed during the current study are not publicly available due to GDPR regulations but are available from the corresponding author on reasonable request.

## Abbreviations

PMT: Protection Motivation Theory
SD: Social distancing
SEM: Socio-Ecological Model
TBP: Theory of Planned Behaviour

## References

[1] Shereen MA, Khan S, Kazmi A, Bashir N, Siddique R (2020). COVID-19 infection: origin, transmission, and characteristics of human coronaviruses. J Adv Res.

[2] Ferguson N, Laydon D, Gilani GN, Imai N. Report 9: Impact of non-pharmaceutical interventions (NPIs) to reduce COVID19 mortality and healthcare demand. 2020. Available from: https://dsprdpub.cc.ic.ac.uk:8443/handle/10044/1/77482 [cited 2020 Aug 26]

[3] Public Health England. Guidance on social distancing for everyone in the UK - GOV.UK. Available from: https://www.gov.uk/government/publications/covid-19-guidance-on-social-distancing-and-for-vulnerable-people/guidance-on-social-distancing-for-everyone-in-the-uk-and-protecting-older-people-and-vulnerable-adults. [cited 2020 Sep 15].

[4] The Health Protection Regulations 2020. Available from: https://www.legislation.gov.uk/uksi/2020/350/made

[5] Setti L, Passarini F, De Gennaro G, Barbieri P, Perrone MG, Borelli M, et al. Airborne transmission route of covid-19: Why 2 meters/6 feet of inter-personal distance could not be enough. Int J Environ Res Public Health; 2020 17. Available from: https://pubmed.ncbi.nlm.nih.gov/32340347/. MDPI AG [cited 2021 Jan 4] 10.3390/ijerph17082932 PMC7215485 32340347

[6] Nissen K, Krambrich J, Akaberi D, Hoffman T, Ling J, Lundkvist Å, et al. Long-distance airborne dispersal of SARS-CoV-2 in COVID-19 wards. Sci Rep. 2020;10:19589. Available from: https://pubmed.ncbi.nlm.nih.gov/33177563/. 10.1038/s41598-020-76442-2. [cited 2021 Jan 4]. 10.1038/s41598-020-76442-2 PMC7659316 33177563

[7] Public Health England. Daily summary | Coronavirus in the UK [Internet] Available from: https://coronavirus.data.gov.uk/. [cited 2021 Jan 4].

[8] Brauner JM, Mindermann S, Sharma M, Johnston D, Salvatier J, Gavenčiak T, et al. Inferring the effectiveness of government interventions against COVID-19. Science (80- ). 2020;eabd9338. Available from: https://www.sciencemag.org/lookup/doi/10.1126/science.abd9338 [cited 2021 Jan 8] 10.1126/science.abd9338 PMC7877495 33323424

[9] Cabinet Office. Staying alert and safe - GOV.UK. Available from: https://www.gov.uk/government/publications/staying-alert-and-safe-social-distancing/staying-alert-and-safe-social-distancing-after-4-july. [cited 2020 Sep 15]

[10] Independent SAGE. COVID-19: what are the options for the UK? Recommendations for government based on an open and transparent examination of the scientific evidence The Independent SAGE Report Additional contributions from. 2020. Available from: www.independentSAGE.org [cited 2020 Sep 1].

[11] Google. COVID-19 Community Mobility Report. 2020. Available from: https://www.gstatic.com/covid19/mobility/2020-05-16_GB_Mobility_Report_en-GB.pdf [cited 2020 May 22]

[12] HM Government. Social distancing: Changes in transport use (Great Britain). 2020. Available from: https://assets.publishing.service.gov.uk/government/uploads/system/uploads/attachment_data/file/886474/2020-05-20_COVID-19_Press_Conference_Slides.pdf#page=4 [cited 2020 Sep 15]

[13] Cowling BJ, Ng DMW, Ip DKM, Liao Q, Lam WWT, Wu JT (2010). Community psychological and behavioral responses through the first wave of the 2009 influenza a(H1N1) pandemic in Hong Kong. J Infect Dis.

[14] Lin L, Savoia E, Agboola F, Viswanath K (2014). What have we learned about communication inequalities during the H1N1 pandemic: a systematic review of the literature. BMC Public Health.

[15] Wirz C, Schwakopf J, Brossard D, Preprints LDB-O, 2020 U. Self-reported compliance and attitudes about social distancing during the COVID-19 outbreak. 2020. Available from: https://osf.io/bv28d/ [cited 2020 Sep 15]

[16] Honarvar B, Lankarani KB, Kharmandar A, Shaygani F, Zahedroozgar M, Rahmanian Haghighi MR (2020). Knowledge, attitudes, risk perceptions, and practices of adults toward COVID-19: a population and field-based study from Iran. Int J Public Health.

[17] Bish A, Michie S (2010). Demographic and attitudinal determinants of protective behaviours during a pandemic: a review. Br J Health Psychol.

[18] Office for National Statistics. Coronavirus (COVID-19) related deaths by ethnic group, England and Wales. Available from: https://www.ons.gov.uk/peoplepopulationandcommunity/birthsdeathsandmarriages/deaths/articles/coronaviruscovid19relateddeathsbyethnicgroupenglandandwales/2march2020to15may2020. [cited 2020 Aug 26]

[19] Public Health England. Beyond the Data: Understanding the Impact of COVID-19 on BAME Communities. 2020. Available from: https://assets.publishing.service.gov.uk/government/uploads/system/uploads/attachment_data/file/892376/COVID_stakeholder_engagement_synthesis_beyond_the_data.pdf [cited 2020 Sep 17]

[20] Rubin GJ, Amlôt R, Page L, Wessely S. Public perceptions, anxiety, and behaviour change in relation to the swine flu outbreak: cross sectional telephone survey. BMJ. 2009;339(7713). 10.1136/bmj.b2651 PMC2714687 19574308

[21] Raisi-Estabragh Z, McCracken C, Bethell MS, Cooper J, Cooper C, Caulfield MJ (2020). Greater risk of severe COVID-19 in black, Asian and minority ethnic populations is not explained by cardiometabolic, socioeconomic or behavioural factors, or by 25(OH)-vitamin D status: study of 1326 cases from the UK biobank. J Public Health (Bangkok).

[22] Yilmazkuday H (2020). COVID-19 and unequal social distancing across demographic groups. Reg Sci Policy Pract..

[23] Liao Q, Cowling B, Lam WT, Ng MW, Fielding R. Situational Awareness and Health Protective Responses to Pandemic Influenza A (H1N1) in Hong Kong: A Cross-Sectional Study. PLoS One. 2010;5(10):e13350. Ng LFP, editor. 10.1371/journal.pone.0013350 PMC2953514 20967280

[24] van der Weerd W, Timmermans DR, Beaujean DJ, Oudhoff J, van Steenbergen JE (2011). Monitoring the level of government trust, risk perception and intention of the general public to adopt protective measures during the influenza A (H1N1) pandemic in the Netherlands. BMC Public Health..

[25] Prati G, Pietrantoni L, Zani B (2011). Compliance with recommendations for pandemic influenza H1N1 2009: the role of trust and personal beliefs. Health Educ Res.

[26] Sharifirad G, Yarmohammadi P, Sharifabad MA, Rahaei Z. Determination of preventive behaviors for pandemic influenza A/H1N1 based on protection motivation theory among female high school students in Isfahan, Iran. J Educ Health Promot. 2014;3:7. Published online 2014 Feb 21. 10.4103/2277-9531.127556. 10.4103/2277-9531.127556 PMC3977398 24741647

[27] Oosterhoff B, Palmer C. Psychological Correlates of News Monitoring, Social Distancing, Disinfecting, and Hoarding Behaviors among US Adolescents during the COVID-19 Pandemic. PsyArXiv; 2020. Available from: https://psyarxiv.com/rpcy4/ [cited 2020 Aug 26]. 10.1001/jamapediatrics.2020.1876 PMC7325067 32597925

[28] Teasdale E, Yardley L (2011). Understanding responses to government health recommendations: public perceptions of government advice for managing the H1N1 (swine flu) influenza pandemic. Patient Educ Couns.

[29] Aburto N, Pevzner E, RL-R-A journal of, 2010 U (2010). Knowledge and adoption of community mitigation efforts in Mexico during the 2009 H1N1 pandemic. Am J Prev Med.

[30] Zhong BL, Luo W, Li HM, Zhang QQ, Liu XG, Li WT (2020). Knowledge, attitudes, and practices towards COVID-19 among chinese residents during the rapid rise period of the COVID-19 outbreak: a quick online cross-sectional survey. Int J Biol Sci.

[31] Kooistra EB, Folmer CR, Kuiper ME, Malouke Esra and Olthuis E, Brownlee M, Fine A, van Rooij B. Mitigating COVID-19 in a Nationally Representative UK Sample: Personal Abilities and Obligation to Obey the Law Shape Compliance with Mitigation Measures (May 11, 2020). Amsterdam Law School Research Paper No. 2020-19, General Subserie Research Paper No. 2020-01. Available at SSRN: https://ssrn.com/abstract=3598221 or 10.2139/ssrn.3598221.

[32] Davis MDM, Stephenson N, Lohm D, Waller E, Flowers P. Beyond resistance: social factors in the general public response to pandemic influenza. BMC Public Health. 2015;15(1). 10.1186/s12889-015-1756-8 PMC4419473 25926035

[33] Andersen M. Early Evidence on Social Distancing in Response to COVID-19 in the United States. 2020. Available at SSRN: https://ssrn.com/abstract=3569368 or 10.2139/ssrn.3569368.

[34] Barrios JM, Hochberg Y V. Risk Perception Through the Lens of Politics in the Time of the COVID-19 Pandemic RISK. 2020. Available from: https://www.nber.org/papers/w27008 [cited 2020 Aug 26]

[35] Painter M, Qiu T. Political Beliefs affect Compliance with COVID-19 Social Distancing Orders (July 3, 2020). Available at SSRN: https://ssrn.com/abstract=3569098 or 10.2139/ssrn.3569098.

[36] Rogers RW (1975). A protection motivation theory of fear appeals and attitude Change1. J Psychol.

[37] Ajzen I, Kuhl J, Beckmann J (1985). From intentions to actions: a theory of planned behavior. Action control: from cognition to behavior.

[38] Mcleroy K, Bibeau DL, Steckler A, Glanz K (1988). An ecology perspective on Health promotion programs. Health Educ Q.

[39] Horne R, Weinman J, Barber N, Elliott R, Morgan M. Concordance, adherence and compliance in medicine taking. 2005. Available from: https://d1wqtxts1xzle7.cloudfront.net/48441294/Concordance_Adherence_and_Compliance_in_20160830-5901-19sk9e2.pdf?1472573380=&response-content-disposition=inline%3B+filename%3DConcordance_adherence_and_compliance_in.pdf&Expires=1600374802&Signature=RiDVScR [cited 2020 Sep 15]

[40] Office for National Statistics. All data related to Population estimates for the UK, England and Wales, Scotland and Northern Ireland: mid-2018. Available from: https://www.ons.gov.uk/peoplepopulationandcommunity/populationandmigration/populationestimates/bulletins/annualmidyearpopulationestimates/mid2018/relateddata. [cited 2020 Sep 16]

[41] Cochran WG (1963). Sampling Techniques.

[42] JISC. Online surveys. Available from: https://www.onlinesurveys.ac.uk/. [cited 2020 Sep 16]

[43] Office for National Statistics. Testing the census. Available from: https://www.ons.gov.uk/census/censustransformationprogramme/testingthecensus. [cited 2020 Aug 26]

[44] Ministry of Housing Communities and Local Government. English indices of deprivation 2019: Postcode Lookup. Available from: http://imd-by-postcode.opendatacommunities.org/imd/2019. [cited 2020 Aug 26]

[45] Gregory TA, Wilson C, Duncan A, Turnbull D, Cole SR, Young G (2011). Demographic, social cognitive and social ecological predictors of intention and participation in screening for colorectal cancer. BMC Public Health..

[46] Tavakol M, Dennick R (2011). Making sense of Cronbach’s alpha. Int J Med Educ.

[47] Francis JJ, Eccles MP, Johnston M, Walker A, Grimshaw J, Foy R (2004). Theory of Planned Behaviour Questionnaires: Manual for Researchers.

[48] Zimet GD, Dahlem NW, Zimet SG, Farley GK (1988). The multidimensional scale of perceived social support. J Pers Assess.

[49] Curtin R, Presser S, Singer E. The Effects of Response Rate Changes on the Index of Consumer Sentiment. Public Opin Q. 2000;64(4):413–428. Available from: https://academic.oup.com/poq/article-lookup/doi/10.1086/318638 [cited 2021 Jan 3] 10.1086/318638 11171024

[50] Singer E, Van Hoewyk J, Maher MP. Experiments with Incentives in Telephone Surveys. Public Opin Q. 2000;64(2):171–188. Available from: https://academic.oup.com/poq/article-lookup/doi/10.1086/317761 [cited 2021 Jan 3] 10.1086/317761 10984332

[51] Moore DL, Tarnai J, Groves RM, Dillman DA, Eltinge JL, Little RJA (2002). Evaluating nonresponse error in mail surveys. Survey Nonresponse.

[52] Smith G. Does gender influence online survey participation?: A record-linkage analysis of university faculty online survey response behavior. scholarworks.sjsu.edu. 2008. Available from: https://scholarworks.sjsu.edu/elementary_ed_pub [cited 2021 Jan 3]

[53] Tu SH, Liao PS. Social distance, respondent cooperation and item nonresponse in sex survey. Qual Quant. 2007;41(2):177–199. Available from: https://link.springer.com/article/10.1007/s11135-007-9088-0 [cited 2021 Jan 3]

[54] Office for National Statistics. Regional ethnic diversity - GOV.UK Ethnicity facts and figures. 2020. Available from: https://www.ethnicity-facts-figures.service.gov.uk/uk-population-by-ethnicity/national-and-regional-populations/regional-ethnic-diversity/latest [cited 2021 Jan 3]

[55] Kim HJ, Fredriksen-Goldsen KI. Nonresponse to a question on self-identified sexual orientation in a public health survey and its relationship to race and ethnicity, Am J Public Health; 2013 103. p. 67–69. Available from: http://ajph.aphapublications.org/. American Public Health Association [cited 2021 Jan 3] 10.2105/AJPH.2012.300835 PMC3518335 23153153

[56] Ahlmark N, Algren MH, Holmberg T, Norredam ML, Nielsen SS, Blom AB, et al. Survey nonresponse among ethnic minorities in a national health survey- A mixed-method study of participation, barriers, and potentials.Ethn Health; 2015 20. p. 611–632. Available from: https://www.tandfonline.com/doi/abs/10.1080/13557858.2014.979768. Routledge [cited 2021 Jan 3] 10.1080/13557858.2014.979768 25411892

[57] Treweek S, Forouhi NG, Narayan KMV, Khunti K (2020). COVID-19 and ethnicity: who will research results apply to?. Lancet.

[58] Smith LE, Amlôt R, Lambert H, Oliver I, Robin C, Yardley L (2020). Factors associated with adherence to self-isolation and lockdown measures in the UK; a cross-sectional survey. Public Health.

[59] Harper CA, Satchell LP, Fido D, Latzman RD. Functional fear predicts public health compliance in the COVID-19 pandemic. Int J Ment Heal Addict. 2020:1-4. 10.1007/s11469-020-00281-5. 10.1007/s11469-020-00281-5 PMC7185265 32346359

[60] Wang C, Chudzicka-Czupała A, Grabowski D, Pan R, Adamus K, Wan X, et al. The Association Between Physical and Mental Health and Face Mask Use During the COVID-19 Pandemic: A Comparison of Two Countries With Different Views and Practices. Front Psychiatry. 2020;11:569981. Available from: https://www.frontiersin.org/article/10.3389/fpsyt.2020.569981/full [cited 2021 Jan 5] 10.3389/fpsyt.2020.569981 PMC7510452 33033485

[61] Jones NR, Qureshi ZU, Temple RJ, Larwood JPJ, Greenhalgh T, Bourouiba L. Two metres or one: what is the evidence for physical distancing in covid-19? BMJ. 2020;370:m3223. Available from: 10.1136/bmj.m3223http://www.bmj.com/ [cited 2021 Jan 8] 10.1136/bmj.m3223 32843355

[62] Brooks SK, Webster RK, Smith LE, Woodland L, Wessely S, Greenberg N (2020). The psychological impact of quarantine and how to reduce it: rapid review of the evidence. Lancet..

[63] Pfattheicher S, Nockur L, Böhm R, Sassenrath C, Petersen MB. The emotional path to action: Empathy promotes physical distancing and wearing face masks during the COVID-19 pandemic. 2020. Available from: https://psyarxiv.com/y2cg5/ [cited 2020 Sep 15] 10.1177/0956797620964422 32993455

[64] Suppes P (1964). On an example of unpredictability in human behavior. Philos Sci.

[65] Cziko GA (1989). Unpredictability and indeterminism in human behavior: arguments and implications for educational research. Educ Res.

[66] Hill EM, Ross LT, Low BS (1997). The role of future unpredictability in human risk-taking. Hum Nat.

[67] Spencer SJ, Josephs RA, Steele CM, Baumeister RF (1993). Low self-esteem: the uphill struggle for self-integrity. Self-esteem: the puzzle of Low self-regard.

