# Supplementary material for: Factors associated with non-adherence to social distancing rules during the COVID-19 pandemic: a logistic regression analysis
Source: BMC Public Health. 2021 Feb 13;21:352. doi: 10.1186/s12889-021-10379-7 (PMC7881344; doi:10.1186/s12889-021-10379-7)
Supplement: Supplementary file 1 — Additional file 1: Digital questionnaire used. [file 12889_2021_10379_MOESM1_ESM.pdf]

COVID-19 Social Distancing & Self-Isolating Study

About You and Your Situation

What is your gender? ✱ *Required*

- Male
- Female
- Other

What is your age in years? ✱ *Required*

Please enter a whole number (integer).

What is your ethnic group? ✱ *Required*

- White
- Mixed
- Asian/Asian British
- Black/Black British
- Other

Is English your first language? ✱ *Required*

- Yes
- No

What is your religion? ✱ *Required*

- Christian
- Buddhist
- Hindu
- Jewish
- Muslim
- Sikh
- Other
- No religion

What is your highest level of education? ✱ *Required*

- No qualifications
- GCSEs or equivalent
- A Levels or equivalent
- Vocational/work-related qualification
- Bachelors degree
- Professional qualification
- Masters degree
- Doctoral degree

What is your postcode? ✱ *Required*

In the most recent general election, who did you vote for? ✱ *Required*

- Conservatives
- Labour
- Lib Dem
- Other
- Did not vote

Today, what is your employment status? ✱ *Required*

- Working as an employee in my normal place of work (not home)
- Working as an employee from home
- Self-employed or freelance in my normal place of work (not home)
- Self-employed or freelance from home
- A furloughed employee
- Unemployed
- Retired
- A student
- Looking after home or family
- Long-term sick or disabled
- Other

As defined by the UK Government, are you a key or essential worker? ✱ *Required*

- Yes
- No

As defined by the UK Government, do you have a medical condition that makes you more vulnerable to coronavirus (COVID-19) or are you pregnant? ✱ *Required*

- Yes
- No

What is your living situation? ✱ *Required*

- I live in a home I own
- I live in a home I rent
- I live in a rented room in a house of multiple occupancy

How many people live at your home? ✱ *Required*

Please enter a whole number (integer).

Do you live with someone who is considered to be more vulnerable to coronavirus (COVID-19), such as someone over 70 years old, someone who has a health condition which makes them more vulnerable, or someone who is pregnant? ✱ *Required*

- Yes
- No

Your Knowledge of Coronavirus (COVID-19)

Coronavirus (COVID-19) can only be caught from a person who has symptoms ✱ *Required*

- True
- False
- Don't Know

Coronavirus (COVID-19) can be spread when an infected person speaks ✱ *Required*

- True
- False
- Don't Know

The most common symptoms of coronavirus (COVID-19) are a dry cough, fever and loss of taste ✱ *Required*

- True
- False
- Don't Know

Coronavirus (COVID-19) can be caught by touching a contaminated surface and then your face ✱ *Required*

- True
- False
- Don't Know

Wearing latex gloves is more effective than hand washing at protecting against coronavirus (COVID-19) ✱ *Required*

- True
- False
- Don't Know

Social distancing restrictions permit me to go outside to walk my dog ✱ *Required*

- True
- False
- Don't Know

If I develop symptoms of coronavirus (COVID-19) I am still permitted to leave my house to collect a medical prescription ✱ *Required*

- True
- False
- Don't Know

If I develop symptoms of coronavirus (COVID-19), I should self-isolate for at least seven days ✱ *Required*

- True
- False
- Don't Know

If someone I live with develops symptoms of coronavirus (COVID-19), but I do not, I should

self-isolate for at least seven days ✱ *Required*

- True
- False
- Don't Know

If I was infected and there was no social distancing, it is estimated that a month later I would have infected over 400 people ✱ *Required*

- True
- False
- Don't Know

Your Social Distancing

In the past two weeks, how many times have you gone out for medication and come within two metres (approx. 3 steps) of someone (e.g., pharmacist, other customers) you don’t live with? ✱ *Required*

Please enter a whole number (integer).

In the past two weeks, how many times have you gone out for groceries and come within two metres (approx. 3 steps) of someone (e.g. cashier, other shoppers) you don’t live with? ✱ *Required*

Please enter a whole number (integer).

In the past two weeks, how many times have you gone out to enjoy parks or public spaces or for exercise and come within two metres (approx. 3 steps) of someone you don’t live with? ✱ *Required*

Please enter a whole number (integer).

In the past two weeks, how many times have you broken social distancing rules to meet with extended family members that don't live with you? ✱ *Required*

Please enter a whole number (integer).

In the past two weeks, how many times have you broken social distancing rules to meet friends that don’t live with you? ✱ *Required*

Please enter a whole number (integer).

In the past two weeks, how many times have you gone out for reasons other than to work, to buy groceries, for medical reasons (e.g. to collect a prescription) to enjoy parks or public spaces or to exercise? ✱ *Required*

Please enter a whole number (integer).

Your Self-Isolating

Have you developed symptoms of coronavirus at any point (even if you believe that these symptoms were not in fact coronavirus)? ✱ *Required*

- Yes
- No

Your Self-Isolating

After developing symptoms of coronavirus, how many times did you leave your house (going to your garden does not count as leaving your house) for any reason within 7 days of developing symptoms? ✱ *Required*

Please enter a whole number (integer).

After developing symptoms of coronavirus, how many times did you have family or friends visit within 7 days of developing symptoms? ✱ *Required*

Please enter a whole number (integer).

Your Self-Isolating With Someone Vulnerable

Have you developed symptoms of coronavirus at any point (even if you believe that these symptoms were not in fact coronavirus) whilst living with someone considered more vulnerable to coronavirus (e.g. over 70, underlying health conditions, pregnant)? ✱ *Required*

- Yes
- No

Your Self-Isolating With Someone Vulnerable

Did you arrange for them to stay with friends or family for 14 days? ✱ *Required*

- Yes
- No

Were you able to maintain a distance of two metres (approx. 3 steps) from them for 7 days? ✱ *Required*

- Yes
- No
- Not relevant; we were able to arrange for them to stay with friends or family

Your Self-Isolating When Someone You Live With Has Symptoms

Has someone you live with developed symptoms of coronavirus at any point (even if they believe that these symptoms were not in fact coronavirus)? * *Required*

- Yes
- No

Your Self-Isolating When Someone You Live With Has Symptoms

After someone you live with developed symptoms of coronavirus, how many times did you leave your house (going to your garden does not count as leaving your house) for any reason within 14 days of developing symptoms? ✱ *Required*

Please enter a whole number (integer).

After someone you live with developed symptoms of coronavirus, how many times did you have family or friends visit within 14 days of developing symptoms? ✱ *Required*

Please enter a whole number (integer).

Your Views

To what extent do you agree with the following statements? ✱ *Required*

	Entirely Disagree	Mostly Disagree	Somewhat Disagree	Neither Agree nor Disagree	Somewhat Agree	Mostly Agree	Entirely Agree
I take care of my own and my family's needs before I worry about the needs of others	☐	☐	☐	☐	☐	☐	☐
Before I act, I think about how my actions might have a negative effect on others	☐	☐	☐	☐	☐	☐	☐
I do what I want, regardless of what others want me to do	☐	☐	☐	☐	☐	☐	☐

Your Beliefs About Coronavirus (COVID-19)

To what extent do you agree with the following statements? ✱ *Required*

	Entirely Disagree	Mostly Disagree	Somewhat Disagree	Neither Agree not Disagree	Somewhat Agree	Mostly Agree	Entirely Agree
There is a good chance that I will get coronavirus (COVID-19)	☐	☐	☐	☐	☐	☐	☐
I do not worry a lot about getting coronavirus (COVID-19)	☐	☐	☐	☐	☐	☐	☐
I'm worried that if I caught coronavirus (COVID-19) my symptoms would be severe	☐	☐	☐	☐	☐	☐	☐

To what extent do you agree with the following statements? ✱ *Required*

	Entirely Disagree	Mostly Disagree	Somewhat Disagree	Neither Agree not Disagree	Somewhat Agree	Mostly Agree	Entirely Agree

I trust the UK Government in their response to COVID-19	☐	☐	☐	☐	☐	☐	☐
I trust that the Government is following the best scientific advice	☐	☐	☐	☐	☐	☐	☐
I trust the scientific advice that the Government has taken	☐	☐	☐	☐	☐	☐	☐

Your Beliefs About Social Distancing

To what extent do you agree with the following statements? ✱ *Required*

	Entirely Disagree	Mostly Disagree	Somewhat Disagree	Neither Agree not Disagree	Somewhat Agree	Mostly Agree	Entirely Agree
I will only leave my home for permitted reasons for as long as the lockdown measures are in place	☐	☐	☐	☐	☐	☐	☐
I will avoid contact with people I do not live with for as long as social distancing restrictions are in place	☐	☐	☐	☐	☐	☐	☐
I will not see friends or extended family in person for as long as the lockdown measures are in place	☐	☐	☐	☐	☐	☐	☐

To what extent do you agree with the following statements? ✱ *Required*

	Entirely Disagree	Mostly Disagree	Somewhat Disagree	Neither Agree not Disagree	Somewhat Agree	Mostly Agree	Entirely Agree
During lockdown, I do not need to leave my home if I don't want to	☐	☐	☐	☐	☐	☐	☐
When I go out for permitted reasons, I cannot stop others from coming within two metres of me	☐	☐	☐	☐	☐	☐	☐
I have responsibilities (e.g., work, childcare) for which I cannot avoid coming into contact with others that I do not live with	☐	☐	☐	☐	☐	☐	☐

To what extent do you agree with the following statements? ✱ *Required*

	Entirely Disagree	Mostly Disagree	Somewhat Disagree	Neither Agree not Disagree	Somewhat Agree	Mostly Agree	Entirely Agree

My family support staying at home and social distancing	☐	☐	☐	☐	☐	☐	☐
My friends are keen to meet up in person, despite the lockdown	☐	☐	☐	☐	☐	☐	☐
I see my neighbours keeping social distancing rules when they are out in my street	☐	☐	☐	☐	☐	☐	☐

Your Beliefs About Self-Isolating

To what extent do you agree with the following statements? ✱ *Required*

	Entirely Disagree	Mostly Disagree	Somewhat Disagree	Neither Agree not Disagree	Somewhat Agree	Mostly Agree	Entirely Agree
If I had symptoms, I would not need to leave my home for any reason	☐	☐	☐	☐	☐	☐	☐
If someone I live with had symptoms, I would not need to leave my home for any reason	☐	☐	☐	☐	☐	☐	☐
I have responsibilities (e.g., work, childcare) for which I would need to leave my home even if I had symptoms	☐	☐	☐	☐	☐	☐	☐

To what extent do you agree with the following statements? ✱ *Required*

	Entirely Disagree	Mostly Disagree	Somewhat Disagree	Neither Agree not Disagree	Somewhat Agree	Mostly Agree	Entirely Agree

My immediate family would not go out for any reason if they had coronavirus symptoms	☐	☐	☐	☐	☐	☐	☐
My friends would not go out for any reason if they had coronavirus symptoms	☐	☐	☐	☐	☐	☐	☐
My extended family would not go out for any reason if they had coronavirus symptoms	☐	☐	☐	☐	☐	☐	☐

Your Support

To what extent do you agree with the following statements? ✱ *Required*

	Entirely Disagree	Mostly Disagree	Somewhat Disagree	Neither Agree not Disagree	Somewhat Agree	Mostly Agree	Entirely Agree
During lockdown, there is a special person who is around when I am in need	☐	☐	☐	☐	☐	☐	☐
During lockdown, there is a special person with whom I can share joys and sorrows	☐	☐	☐	☐	☐	☐	☐
During lockdown, my family really tries to help me	☐	☐	☐	☐	☐	☐	☐
During lockdown, I get the emotional help & support I need from my family.	☐	☐	☐	☐	☐	☐	☐

During lockdown, I have a special person who is a real source of comfort to me							
During lockdown, my friends really try to help me							
During lockdown, I can count on my friends when things go wrong							
During lockdown, I can talk about my problems with my family							
During lockdown, I have friends with whom I can share my joys and sorrows							

During lockdown, there is a special person in my life who cares about my feelings	☐	☐	☐	☐	☐	☐	☐
During lockdown, my family is willing to help me make decisions	☐	☐	☐	☐	☐	☐	☐
During lockdown, I can talk about my problems with my friends	☐	☐	☐	☐	☐	☐	☐

During lockdown, are you getting the help you need with your financial situation from UK Government schemes? ✱ *Required*

- ○ Yes
- ○ No
- ○ Help Not Needed

During lockdown, are you getting the help you need with your housing situation from UK Government schemes? ✱ *Required*

- ○ Yes
- ○ No
- ○ Help Not Needed

During lockdown, are you getting the help you need from local community services (e.g., your council, voluntary or charity organisations)? ✱ *Required*

- ◯ Yes
- ◯ No
- ◯ Help Not Needed

# Email Address & Future Involvement

If you are randonly selected to win a £100 Aldi voucher, we need your email address to notify you. Providing your email address is optional.

What is your email address? *Optional*

More info

Please enter a valid email address.

We would like to conduct follow-up telephone interviews with certain individuals. This involvement is optional and does not impact your standing in the proze draw for the £100 Aldi vouchers.

I am willing to be contacted by email to schedule a follow up telephone interview for which I will be compensated with a £20 Aldi voucher ✱ *Required*

- Yes
- No

Thank You

This is the end of the questionnaire.

Thank you for completing it and for participating in this research project.

If you are randomly selected to win a £100 Aldi voucher we will notify you via email.
